# Impact of Nursing Practice Environments in Work Engagement and Burnout: A Systematic Review

**DOI:** 10.3390/healthcare13070779

**Published:** 2025-03-31

**Authors:** Virginia Chiminelli-Tomás, Verónica Tutte-Vallarino, Augusto Ferreira-Umpiérrez, Juan José Hernández-Morante, Cristina Reche-García

**Affiliations:** 1 Department of Health and Wellness, Universidad Católica del Uruguay, Montevideo 11600, Uruguay; vchiminell@ucu.edu.uy (V.C.-T.); vtutte@ucu.edu.uy (V.T.-V.); auferrei@ucu.edu.uy (A.F.-U.); 2 Eating Disorders Research Unit, Universidad Católica de Murcia, Guadalupe, 30107 Murcia, Spain; jjhernandez@ucam.edu; 3 Multidisciplinary Research Group on Health Psychology, Universidad Católica de Murcia, Guadalupe, 30107 Murcia, Spain

**Keywords:** PES-NWI, work engagement, burnout, work environment, nursing

## Abstract

**Background/Objectives**: Work environment greatly affects nursing activities. This study aimed to explore the impact of the professional nursing environment on the risk of burnout symptomatology and work engagement (a burnout-moderating variable). **Methods**: A systematic review has been carried out. Selection criteria included cross-sectional studies that evaluated the professional nursing environment with the PES-NWI scale and also evaluated the levels of burnout and/or engagement in nurses. The PubMed, Scopus, Cinahl and WoS databases have been reviewed until November 2024, and potential articles manually selected by two researchers independently. **Results**: Eighty-four articles were selected, and after applying selection and exclusion criteria, 14 papers were finally included. Most studies were conducted in Europe. The quality evaluation was carried out using the JBI critical appraisal checklist. All retrieved studies focused on burnout, and only two works also evaluated work engagement. **Conclusions**: Overall, the trend indicates that a favorable professional environment was related to lower burnout symptoms, lower intentions to leave work and higher levels of work engagement. In addition, an adequate work environment was associated with less stress and higher quality of care, social support, professional development, leadership, nurse–doctor collaboration, nurse participation in hospital issues, staffing and job satisfaction.

## 1. Introduction

Nurses’ shortage has been identified as a global public health crisis, with projections indicating a deficit of 4.5 million professionals by 2030 [1,2]. This phenomenon puts the sustainability of health systems at risk, especially in contexts where the demand for health care continues to increase due to population aging [3] and the growth in the prevalence of chronic diseases requires a large amount of Health Services resources [2]. Various studies have noted that working conditions, along with factors such as an aged nurse workforce, have exacerbated this deficit [4].

The COVID-19 pandemic aggravated this situation, exposing nursing professionals to a greater workload and extreme working conditions, which has increased burnout levels. Burnout, characterized by emotional exhaustion and depersonalization, has been shown, in the context of nursing, to be a key factor in the intention to leave the profession [5]. In contrast, engagement, defined as a state of high energy and appointment to work, has emerged as an antidote to burnout, promoting job satisfaction and staff engagement [6].

In this line, the conditions of the work environment play a fundamental role in the prevalence of burnout and engagement. According to a recent systematic review, job demands, such as work overload, shift types, the concurrence of negative events and the type of service, as well as personal and organizational resources, such as social support, psychological capital and the possibility of expressing emotions, can influence the levels of both burnout and engagement in nursing professionals [7].

The concept of the “magnetic hospital”, developed in the 1980s in the United States, has been a successful model for creating work environments that optimize both engagement and the prevention of burnout [8]. At these hospitals, nurses report higher job satisfaction and better health outcomes for patients. These findings reinforce the need to create work environments that not only minimize sources of stress but also strengthen resources that promote professional well-being.

The Practice Environment Scale of the Nursing Work Index (PES-NWI) is one of the most widely used tools to assess these work environments and has been shown to be effective in measuring five key dimensions: participation in hospital affairs, staffing, leadership support, quality of care and interprofessional relationships [9]. Various international studies have validated the PES-NWI in different contexts, demonstrating its usefulness for measuring the nursing work environment. In Spain, Brazil and Colombia, this scale has been successfully adapted and validated, showing consistency in the evaluation of environments of nursing work [10,11,12].

Despite the evaluation of the PES-NWI in different social contexts, there is a lack of comprehensive evaluation of these previous studies, which limits the extrapolation of the results of these previous works. Therefore, the aim of this systematic review was to analyze the available evidence on the role of the professional practice environment (assessed with the PES-NWI) in burnout symptoms and engagement in nursing, which would help identify protective factors for burnout related to work engagement and other factors.

## 2. Materials and Methods

The PRISMA (Preferred Reporting Items for Systematic reviews and Meta-Analyses) protocol has been followed. The present systematic review was registered in Open Science Framework Registries (#protocol: https://doi.org/10.17605/OSF.IO/Z2V5S).

### 2.1. Data Sources and Literature Search

The PubMed database was searched for suitable articles published up to 30 November 2024. The following MeSH (Medical Subject Headings) search terms were used (“PES-NWI” [All Fields] AND “burnout OR engagement” [All Fields] AND “nurs*” [All Fields]) [MeSH Terms]. Title, abstract and keywords were carefully examined to identify relevant papers. A parallel search in the ISI Web of Knowledge and the Scopus and Cinahl databases was performed to check for additional papers using the same terms. Reference lists of identified manuscripts and reviews were checked manually.

### 2.2. Eligibility Criteria

The PICOS criteria for defining the research questions are shown in Table 1 Studies that met the following criteria were eligible: participants were nursing staff; intervention included hospital settings assessed by the PES-NWI; no comparative intervention; study design included burnout and/or engagement outcome measures in observational studies. Articles in English were accepted. There were no date limits. Gray literature, reviews, studies that related the nursing environment to variables other than burnout and engagement (or to none of them) and qualitative studies were excluded.

### 2.3. Selection Process and Data Extraction

Two researchers (C.R. and V.C.) autonomously evaluated the titles and abstracts of all retrieved papers to find those that met the eligibility criteria. The full texts of eligible studies were retrieved and independently assessed by the same investigator. Disagreements were resolved by discussion, and a consensus was made.

The data for all retrieved works were collected by one author (C.R.) and confirmed by another author (V.C.). The extracted data included study type, population and participant characteristics, study information and assessment of PES-NWI and burnout or engagement. Disagreements were identified and resolved by consensus.

### 2.4. Quality Assessment

Two authors, C.R. and V.C., assessed the quality of the fourteen included studies twice, using a checklist for analytical cross-sectional studies in JBI Systematic Reviews. The JBI is an international research organization based at the Faculty of Medical and Health Sciences, University of Adelaide, South Australia [13].

Conflicts were resolved by discussion. The risk of bias tool rates the following domains: were the criteria for inclusion in the sample clearly defined?; were the study subjects and the setting described in detail?; was the exposure measured in a valid and reliable way?; were objective, standard criteria used to measure the condition?; were confounding factors identified?; were strategies to deal with confounding factors stated?; were the outcomes measured in a valid and reliable way?; was appropriate statistical analysis used?; and Overall appraisal.

**Figure 1:**
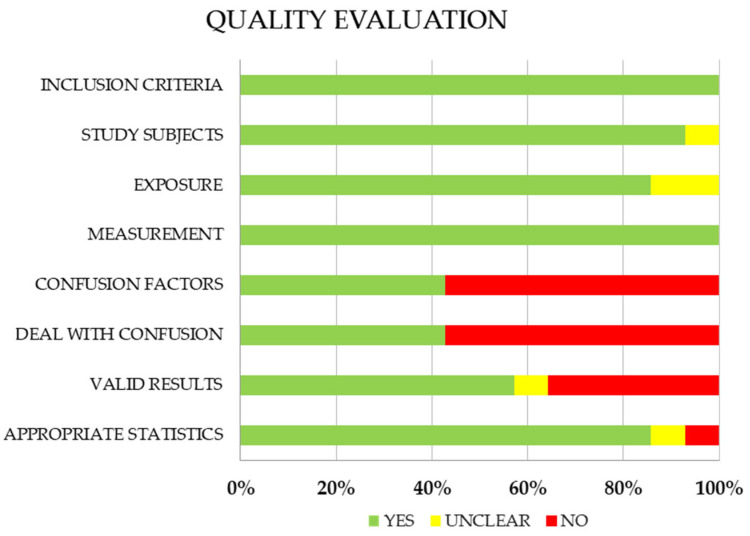
Quality assessment of the fourteen studies included in the revision.

We found that 100% of the studies present clearly defined inclusion criteria, gave a detailed description of the study participants and the environment and clearly outlined objective criteria for measuring the condition. In the case of the evaluation of exposure measured in a valid and reliable way, a study appeared that provided little clarity. 67% of the confounding factors were not identified, and strategies to deal with the confounding factors were not established. 42% of the results were not measured in a valid and reliable way. Finally, the statistical analyses were mostly adequate (83%). The general assessment of the studies allowed for the inclusion of all of them (Figure 1).

## 3. Results

### Search Results

**Figure 2:**
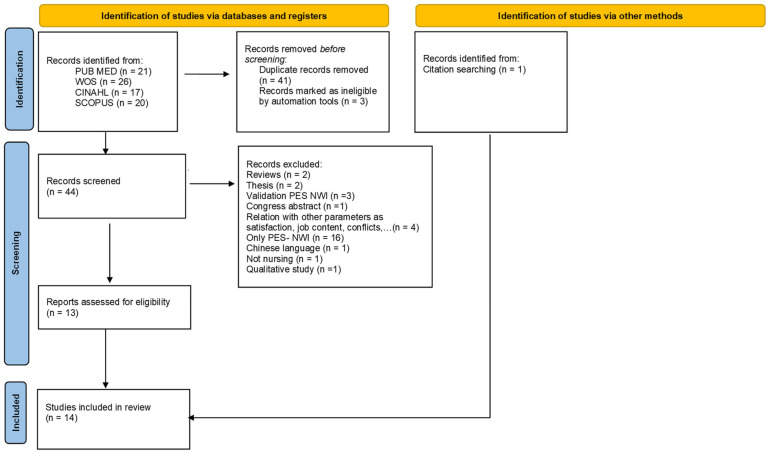
Flow diagram of the literature search process.

The initial literature search identified 84 potentially eligible studies (Figure 2). After title and abstract review, 44 studies were discarded. Duplicates were also removed. The remaining 44 manuscripts were carefully reviewed. Ultimately, 14 studies met the inclusion criteria for this systematic review.

All studies used a cross-sectional design. A detailed overview of studies that investigated effects and reviewed the available evidence on the professional practice environment (assessed with the PES-NWI) in burnout symptoms and engagement in nursing is provided in Table 2.

Most studies were conducted in Europe (n = 8; 57.1%) and were published between 2006 and 2023 with an increasing trend over time. Six of the studies were from Spain, two from China, two from Poland, one from Belgium, one from New Zealand, one from the Philippines, and one from Canada. All studies focused on burnout (n = 14; 100%), and only two evaluated engagement (n = 2; 14.2%). The 14 studies that examined the PES-NWI included a total of 31,919 adult nursing participants, mostly women whose age ranged from 22 to 59 years.

All studies reviewed used the Practice Environment Scale-Nursing Work Index (PES-NWI) to evaluate the professional practice environment; 14 papers employed the Maslach burnout inventory human services survey (MBI-HSS); two works used the Utrecht Work Engagement Scale (UWES) to assess work engagement as well as the Perceived Stress Scale (PSS-5) to evaluate stress. In other cases, each instrument was used in a single study.

The results derived from the literature indicate that a favorable professional practice environment was related to lower burnout symptoms, lower intentions to leave work [5,14,15,16] and higher levels of work engagement [17], as well as with less stress and higher quality of care [15].

Individual factors that protected unfavorable perceptions of the work environment were work experience [14,18], advanced age [14] and job satisfaction [16]. The organizational factors that allowed for good evaluations of the nursing practice environment were day shifts [19], the possibility of professional development [18], nursing leadership [20], collaborative relationships between nurse and doctor [16], the foundations of nursing for quality care [16,21], the participation of nurses in hospital issues [16,17,21] and staffing [5,14,21,22,23]. Staffing is the most studied variable that modulates in the professional practice environment in nursing. Moreover, social support favored nursing engagement and could moderate the relationship between work demands and burnout [17].

Kind nursing services had different perceived professional environments [24], an issue that depended on the size of the hospital. The smaller health centers showed the most positive working conditions [23]. Lower scores were found among university nurses compared to graduate nurses in the perception of the nursing practice environment and in work engagement [18].

**Table 2:** Studies selected for systematic review.

Reference	Population and Participant Characteristics	Instruments	Results
Bruyneel et al. (2023) [5]. Belgium	N = 2321 (74.5% women); average age 35. Descriptive cross-sectional observational design	Participant characteristics questionnaire; of the organization (PES-NWI), burnout (MBI-HSS), intention to leave the hospital and/or the profession (single question) Nursing staffing questionnaire	High prevalence of high risk of burnout and intention to leave work and profession after COVID-19 in Belgium with differences depending on the hospital environment and patient-to-nurse ratios.
Yuchun-Yang et al. (2023) [18]. China	N = 199 (92.5% women); average age 28.3. Descriptive cross-sectional observational design	Nursing Career Scale, organizational scale (PES-NWI), burnout (MBI-HSS), Utrecht Work Engagement Scale (UWES)	Most Japanese nurses with university degrees obtained lower scores on the PES-NWI Scale and work engagement compared to those with diploma degrees. Furthermore, with more years of experience they showed better results in terms of interpersonal relationships and professional development.
Malinowska- Lipień et al. (2023) [14]. Poland	N = 1509 (98.32% women); mean age 43.99 ± 10.28. Descriptive cross-sectional observational design.	Participant characteristics questionnaire; of the organization (PES-NWI), burnout (MBI-HSS), questionnaire on the subjective assessment of patient safety and quality of care	48.84% declared they wanted to leave their current job. They were the youngest, with the least seniority in the service and in the hospital. Increasing the number of patients by one was associated with a 1% increase in the risk of leaving work. An increase in emotional exhaustion increased the risk of leaving work by 2%
Radosz-Knawa et al. (2022) [22]. Poland	N = 209 (83.7% women); age range 22–56 years. Descriptive cross-sectional observational design	Nursing care rationing (BERNCA-R), Nursing Work Index (PES-NWI), burnout (MBI-HSS)	The limitation of care is correlated with the occurrence of adverse events among patients, with a lower appreciation of working conditions and, in addition, with a greater risk of emotional exhaustion and depersonalization among nursing staff.
Falguera et al. (2020) [15]. Philippines	N = 549 (78% women); mean age 29.80 ± 7.80 Descriptive cross-sectional observational design	Participant characteristics questionnaire; of the organization (PES-NWI); satisfaction (single question), stress (PSS-5), burnout (MBI-HSS) and quality of patient care (single question)	A favorable nursing practice environment presents a significant and negative relationship with burnout and work stress and a positive one with the quality of patient care.
Tabakakis et al. (2020) [25]. New Zealand	N = 480 (94% women); mean age 47.1 ± 12.7. Descriptive cross-sectional observational design	Participant and Organizational Characteristics Questionnaire (PES-NWI), the Copenhagen Burnout Inventory (CBI), Negative Acts Questionnaire-Revised (NAQ-R)	Workplace factors are associated with nursing burnout.
Li-Yuan et al. (2020) [21]. China	N = 1300 (96.0% women), mean age 29.63. Descriptive cross-sectional observational design	Participant characteristics questionnaire; of the organization (PES-NWI), burnout (MBI-HSS)	The nursing practice environment was rated as favorable, in general. Approximately 40% of respondents reported experiencing emotional exhaustion and depersonalization. Nurse burnout was associated with participation in hospital affairs, foundations of nursing care for quality of care and adequate staffing.
Moreno-Casba et al. (2018) [19]. Spain	N = 635; mean age 41 ± 10.03. Descriptive cross-sectional observational design	Participant characteristics questionnaire; of the organization (PES-NWI), burnout (MBI-HSS), Morning-Evening Scale (Horne and Östberg), Daytime Sleepiness Scale (Epworth1), Pittsburgh Sleep Quality Scale (PSQI)	83.7% perceived the quality of care as good/excellent. 55.1% rated the work environment as good/excellent. 39% of hospitals were classified as unfavorable according to the PES-NWI. 15.4% of the nurses had a high level of burnout, and 58.3% had a low level. Sleep quality varied depending on the shift, with poorer quality on night shifts, an issue that affected the safety of care, the work environment and the provision of care.
García-Sierra et al. (2016) [17]. Spain	N = 100 (89.58% women), mean age 40.58. Descriptive cross-sectional observational design	Demands, control and support with the Job Content Questionnaire (JCQ), burnout (MBI-HSS), and work engagement (UWES)	Social support is a significant predictor of nurses’ participation and engagement. Demands are a predictor of nurse burnout. Work engagement moderates the relationship between job demands and burnout.
Escobar-Aguilar et al. (2013) [16]. Spain.	N = 1886 (87.8% women); mean age 40.4 ± 9.9. 1469 patients (47.5% women); mean age 57.8 ± 18.3. (SENECA). N = 2139 (RN4CAST). Descriptive cross-sectional observational design, secondary analysis	Participant characteristics questionnaire; of the organization (PES-NWI), burnout (MBI-HSS)	Institutions with a favorable work environment in the “Relationships” dimensions “nurse-doctor relationship”, “Nursing foundations for quality care”, “Nurse participation in hospital issues” and “job satisfaction” are related to a greater perception of the patients about the safety of care, as well as less emotional exhaustion and depersonalization of professionals.
Fuentelsaz-Gallego et al. (2013) [24]. Spain	N = 5654 (89% women from Medical Surgical Units (UMQ) and 84% from Critical Care Units (CU); mean age in UMC of 37.6 and in UC of 31.1. Descriptive cross-sectional observational design	Organization questionnaire (PES-NWI), burnout (MBI-HSS)	The PES-NWI presented better values in UMQ with the exception of the provision and adequacy of resources. Burnout was higher in the UMQs with 23% of nurses having high values. Job satisfaction was lower in UCs, with 70% of nurses very or moderately satisfied.
Abad-Corpa et al. (2013) [23]. Spain	N = 687 (80.4% women); average age 34.1. Descriptive cross-sectional observational design	Questionnaire on personal characteristics, quality and safety of the patient, the organization (PES-NWI), burnout (MBI-HSS)	The patient/nurse ratio was 11.7 with variability between hospitals. Two hospitals had an unfavorable climate, and three hospitals had a favorable one (large hospitals had worse ratings); a low intention to leave work was observed (16.8%). Regarding burnout, 22% had high levels of emotional exhaustion, 19.3% of depersonalization and 48.7% of personal achievement. The perception of quality showed differences between centers and that of adverse effects was more favorable in small hospitals.
Fuentelsaz-Gallego et al. (2012) [24]. Spain	N = 5654 (88% women); average age 37.5. Descriptive cross-sectional observational design	Participant characteristics questionnaire; of the organization (PES-NWI); satisfaction (single question), stress (PSS-5), burnout (MBI-HSS)	26% indicated that they would like to leave the hospital. 55% of the nurses indicated that they were moderately satisfied with their work. The work environment was unfavorable for 48% of the nurses. 22% had a high level of burnout.
Leiter et al. (2006) [20]. Canada	N = 8597 (97.5% women); average age 44.1. Descriptive cross-sectional observational design. Factor analysis.	Participant characteristics questionnaire; of the organization (PES-NWI), burnout (MBI-HSS)	The areas of work life have a defined relationship with burnout channeled through a path from staffing to burnout and a path from nursing model of attention to personal fulfillment. A model indicates that nursing leadership is fundamental to interventions to improve the quality of work life of nurses.

## 4. Discussion

This review evaluated the impact of the work environment on nurse professional burnout and engagement and confirms the importance of a favorable professional practice environment in promoting mental health and occupational well-being of nurses, as well as its influence on the quality of care provided. The results found from 14 articles that met the inclusion and exclusion criteria allowed the researchers to clarify the relationship between the study variables and the sociodemographic and organizational data which could most influence the possibility of developing burnout or, otherwise, promote engagement.

One requirement that was taken into account to select previous studies was the employment of the PES-NWI to evaluate work environment, which, as commented previously, is considered currently to be the gold-standard method. 93% of the selected works used the MBI as an instrument to measure burnout in human services professionals such as nurses. Likewise, the same percentage prevails in studies that measured the nursing environment through the PES-NWI, which could refer to the potential that these instruments have as tools to assess the relationship between burnout and the organizational context of health centers.

Regarding the geographical distribution of the studies, it reflects a predominance in Europe, especially Spain (six studies), which could be influenced by a greater focus on research on workplace well-being in this context. It could also be a consequence of higher burnout symptomatology in Spain compared to other European countries, probably due to the low patients/nurses ratio in this country.

Likewise, the increasing trend in the number of quality publications suggests greater global awareness about the importance of the nursing work environment and its impact on the quality of care. Undoubtedly, an adequate identification of factors increasing work engagement and reducing burnout may improve the quality of care delivered by nurses and other health professionals.

The data derived from the literature review showed that an adequate work environment is consistently associated with lower levels of burnout [15], balancing labor demands and resources [7], as well as with higher levels of engagement [6].

There is a growing trend toward studying protective factors that facilitate a favorable work environment, compared to previous studies that evaluated risk factors such as burnout. The present review allowed for an identification of protective factors at both individual and organizational levels. At the individual level, job satisfaction, sleep quality and older age emerged as protective variables that positively influence the perception of the work environment [16,23]. In fact, younger nurses with less seniority in the service showed a higher desire to leave their jobs [14], which reinforces the relevance of age regarding job satisfaction. At the organizational level, elements such as adequate staffing, day shifts and nursing leadership were essential to promote a positive work environment, improving the perceived quality of care, motivation [19,20,21] and job satisfaction [14,22].

Other studies also highlighted the importance of interdisciplinary collaboration and nurses’ participation in hospital decision making as essential components of a favorable work environment [16,17]. Moreover, perceptions of the work environment were more positive in smaller facilities, which could suggest that organizational scale could influence work dynamics [23].

In short, the work environment has been shown to predict job engagement [26], through feelings of self-efficacy and achievement motivation [27], and facilitates staff retention and perceived quality of care [28].

These findings highlighted the importance of intervening in working conditions to improve not only individual but also organizational and patient outcomes and highlights the need for organizational strategies that prioritize both reducing burnout and strengthening engagement, considering the protection factors described.

There are arguments about the importance of engagement in the organizational and health context [7]. Recently it has been concluded that organizational engagement correlates with work engagement [29]. In line with recent studies, it is suggested that health policy makers implement strategies to improve the engagement of their nurses, reduce their burnout and stress and foster compassion; to increase the quality of patient care; nurse retention; their job satisfaction and improved organizational results [30].

Overall, there are several implications derived from the results of this review. On the one hand, an improved work environment would reduce burnout and intentions to leave work, which could lead to greater job satisfaction and quality of patient care. On the other hand, a suitable work environment could improve nursing professionals’ engagement and staffing levels. This situation has several implications both for the health system and the nursing profession. On the one hand, the Healthcare System may be improved by enhanced patient care, reduced turnover rates, improved efficiency and better staff collaboration. On the other hand, nurses may increase their job satisfaction, professional growth, mental and emotional well-being and sense of purpose. This is why this review allows us to know the variables to be addressed in the design of a possible future intervention that aims to improve the nursing work environment.

Although the findings are consistent, the limited geographical diversity of the studies and the focus on cross-sectional designs restrict the ability to establish causal relationships. Furthermore, the limited literature on the engagement variable suggests the need to further explore factors that promote the well-being and motivation of nurses, beyond reducing burnout. Regarding the quality of the articles reviewed, the biggest problem is that most articles do not identify possible confounding factors as well as strategies to address them, so it is proposed that this issue be addressed in future studies. It is important to remember that the present systematic review was conducted on observational studies. Despite the growing importance of systematic reviews of observational studies in public health, not all observational studies are of equal evidential value and require careful assessment of risk of bias. Furthermore, their inclusion may increase the resource demands of the review [31].

Finally, we consider it crucial that future research explore work environments in underrepresented regions, especially in low- and middle-income countries, where nursing work challenges may differ significantly with high-income countries. Likewise, longitudinal studies could provide a more robust view of the causal dynamics between the practice environment and its associated outcomes. Finally, integrating blended approaches that combine quantitative and qualitative methods could offer a richer perspective on nurses’ experiences and the nuances of the work environment.

## 5. Conclusions

Improving the work environment can help to alleviate some of the challenges of professional well-being in nursing. Concretely, promoting a favorable professional practice environment may offer significant opportunities to reduce burnout, promote engagement and improve the quality of care in health systems. In fact, the potential benefits of a positive environment include greater job satisfaction, better cross-functional relationships, staff retention, and more sustainable organizational results. These advantages have been well documented and, in some cases, validated in various contexts.

Protective factors for a favorable professional environment were perceived social support, the possibility of professional development, leadership, collaborative relationships between nurses and doctors, nursing fundamentals for quality care, nurse participation in hospital issues and staffing, but also aspects such as day shifts and small hospitals.

However, despite this theoretical and practical potential, improving work environments still faces numerous barriers, such as insufficient staff, excessive workload and the lack of specific strategies to promote engagement. The development of longitudinal and further research in less represented regions will be crucial to overcome these limitations.

## Figures and Tables

**Table 1:** PICOS criteria for inclusion and exclusion of studies.

Parameter	Description
Population	Nurses
Intervention	PES-NWI: professional environment
Comparison	No intervention
Outcome	Engagement and burnout
